# Potential value of cerebrospinal fluid α-synuclein in the identification of postoperative delirium undergoing knee/hip arthroplasty: The perioperative neurocognitive disorder and biomarker lifestyle study

**DOI:** 10.3389/fnins.2022.935869

**Published:** 2022-10-24

**Authors:** Xu Lin, Yuwei Guo, Rui Dong, Bin Wang, Yanlin Bi

**Affiliations:** ^1^Department of Anesthesiology, Qingdao Municipal Hospital Affiliated to Qingdao University, Qingdao, China; ^2^Department of Anesthesiology, Drum Tower Hospital Affiliated to Medical College of Nanjing University, Nanjing, China

**Keywords:** α-synuclein, postoperative delirium, biomarker, cerebrospinal fluid, knee/hip arthroplasty

## Abstract

**Objective:**

Postoperative delirium (POD) is a common postoperative complication, which may be associated with α-synuclein (α-syn). The purpose of this study was to explore the association between the expression level of α-syn in cerebrospinal fluid (CSF) and POD.

**Methods:**

We conducted a prospective observational cohort study, which involved in 740 participants (mean age of 61.86 years, range 40–90 years; 40% female) from the Perioperative Neurocognitive Disorder And Biomarker Lifestyle (PNDABLE) study in the final analysis. POD was diagnosed using the Confusion Assessment Scale (CAM), and its severity was measured using the Memorial Delirium Assessment Scale (MDAS). Enzyme-linked immune-sorbent assay (ELISA) was used to detect the concentrations of α-syn, Aβ40, Aβ42, T-tau, and P-tau in CSF.

**Results:**

The incidence of POD was 11.22% (83/740). The logistic regression analysis showed that the increased concentrations of CSF α-syn (OR = 1.005, 95%CI 1.004–1.006, *P* < 0.001), P-tau (OR = 1.093, 95%CI 1.071–1.115, *P* < 0.001), and T-tau (OR = 1.008, 95%CI 1.006–1.009, *P* < 0.001) were risk factors of POD. Linear regression showed that CSF α-syn had positive correlations with P-tau (β = 0.480, *P* < 0.001), T-tau (β = 0.334, *P* < 0.001), while negative correlations with Aβ40 (β = –0.378 *P* < 0.001), Aβ42 (β = -0.800, *P* = 0.001) in POD patients. Mediation analyses showed the association between α-syn and POD was partially mediated by tau pathologies (proportion: 16–17%).

**Conclusion:**

CSF α-syn is one of the preoperative risk factors for POD, which may be mediated through tau pathologies.

**Clinical trial registration:**

[www.ClinicalTrials.gov], identifier [ChiCTR20 00033439].

## Introduction

Postoperative delirium (POD) is characterized by acute cerebral dysfunction or failure, as well as fluctuating consciousness, accompanied by obvious impairment of attention and cognitive function. POD often occurs between postoperative days 1–7 (or before discharge) (Rudolph and Marcantonio, 2011; Zenilman, 2017). The incidence of POD in surgical patients was 20–45%, and its morbidity and mortality were high. It can prolong the hospitalization of patients, seriously affect patients’ quality of life, and increase the burden on families and society (Sieber and Barnett, 2011; Inouye et al., 2014; Jin et al., 2020). The previous study has shown that biomarkers such as amyloid (Aβ) and Tau protein in cerebrospinal fluid (CSF) have become powerful predictors of POD (Xie et al., 2014). However, the relationship between the expression level of α-syn in CSF and POD is still unclear.

α-synuclein (α-syn) is commonly considered as a synaptic protein, which consists of 140 amino acid residues with a molecular weight of 14 kDa (Burré, 2015). It is mainly distributed in presynaptic terminals of neurons and can also be found in cerebrospinal fluid (CSF) (Guardia-Laguarta et al., 2014). α-syn has biological functions, such as regulating synaptic plasticity and synaptic dopamine content, as well as promoting synaptic vesicle formation (Danzer et al., 2007; Golovko et al., 2009). Meanwhile, several studies have shown that abnormal α-syn can lead to pathological changes, which were associated with POD (Golovko et al., 2009).

The abundant neuroprotein in the brain—α-syn, which is not only involved in the maintenance of normal synaptic function, but also associated with various neurodegenerative diseases (Guo et al., 2020). Studies have shown that α-syn may interact with two key proteins Aβ and tau in the pathogenesis of cognitive impairment, promote their aggregation, aggravate neuronal damage, and accelerate the decline in cognitive function (Herukka et al., 2011; Lambert et al., 2013; Frisoni et al., 2017; Sulzer and Edwards, 2019). Importantly, the accumulation of α-syn has also been shown to significantly interfere with the cognition of mice and associate with synaptic dysfunction in cognitive impairment (Corder et al., 1993; Barnes and Yaffe, 2011; Hebert et al., 2013; Liu et al., 2013; Baumgart et al., 2015).

Since the CSF is directly connected with the extracellular fluid of the brain tissue, which can dynamically reflect the metabolism of the brain tissue and the stability of the internal environment (Olsson et al., 2016), we conducted a large prospective observational cohort study and hypothesized that CSF α-syn was associated with POD. And then three analyses were conducted. Firstly, to evaluate whether CSF α-syn was associated with POD. Secondly, to explore the relationship among a-syn with Aβ40, Aβ42, total tau (T-tau), and phosphorylated tau (P-tau) in CSF. Thirdly, to examine whether the effect of α-syn on POD is mediated by CSF biomarkers in a large sample Chinese adults with normal cognitive function before operation.

## Materials and methods

### The perioperative neurocognitive disorder and biomarker lifestyle study

Perioperative Neurocognitive Disorder’s And Biomarker LifestylE Perioperative Neurocognitive Disorder And Biomarker Lifestyle (PNDABLE) study is an ongoing large-scale cohort study, focusing on the risk factors and biomarkers of perioperative neurocognitive disorder (PND) in the Han population of northern China. The purpose of PNDABLE was to determine the genetic and environmental factors of PND biomarkers, as well as the lifestyle factors that might affect the risk of PND in the non-demented northern Chinese Han population, so as to form the basis for disease prevention and early diagnosis. All participants provided written informed consent, and they were later instructed to withdraw their permission if they changed their mind. Their CSF samples could be used for research purposes in the future. This study (Ethical Committee N°2020 *PRO FORMA* Y number 005) was approved by the Ethical Committee Qingdao Municipal Hospital affiliated to Qingdao University (Chairman Prof Yang) on 21th May 2020, and it also was registered at ClinicalTrials.gov (ChiCTR2000033439) and registered on 01 June 2020, and was carried out in accordance with the Helsinki Declaration.

### Study participants

All participants in the PNDABLE study were Han Chinese aged between 40 and 90. The subjects were 833 patients who underwent total knee/hip arthroplasty under combined spinal and epidural anesthesia in Qingdao Hospital affiliated to Qingdao University from June 2020 to December 2021. The inclusion criteria of this study included: (1) 40–90 years old; (2) Han patients in northern China; (3) American Society of Anesthesiologist’ (ASA) 1–2 scores; (4) good preoperative cognitive status [Mini-Mental State Examination scale (MMSE) ¿ 23 points or Montreal cognitive assessment scale (MoCA) ¿ 26 points] and no language communication barrier; and (5) education level sufficient to complete the preoperative cognitive function test. The exclusion criteria included: (1) central nervous system infection, head trauma, multiple sclerosis, neurodegenerative diseases (such as POD, epilepsy, dementia, Parkinson’s disease), or other major neurological diseases; (2) major psychological disorders; (3) family history of severe systemic diseases that may affect levels of CSF biomarkers such as Aβ and Tau (e.g., malignant tumor); (4) genetic family history; (5) preoperative MMSE ≤ 23 points or MoCA ≤ 26 points; (6) severe visual and hearing impairment; (7) unwilling to abide by the agreement or procedure. All participants underwent clinical and neuropsychological assessments, biochemical tests, and CSF sample collection. Demographic information and medical history were collected by comprehensive questionnaires, electronic medical record systems and laboratory examination management systems.

We interviewed all participants the day before surgery and collected baseline data, including age, gender, body weight, ASA physical status, education level, as well as MMSE and MoCA scores. We collected other information based on the patient’s medical history, including complications and the time from injury to surgery. In addition, patients or their guardians were asked to sign a written informed consent form.

### Neuropsychological testing

Each patient was scheduled to test MMSE and MoCA scores by a neurologist before operation. The assessment of POD was performed at 10 a.m. and 2 p.m. twice a day on the 1–7 days after operation or before discharge. At the same time, Numerical Rating Scale (NRS) score of 0–10 (a lower score indicates a lower degree of pain) was used to assess the patients’ pain (Chung et al., 2016). The presence of POD was defined according to Confusion Assessment Scale (CAM), and the severity of POD was defined according to the Memorial Delirium Assessment Scale (MDAS). The Chinese versions of CAM and MDAS have been proved to have good reliability and validity in the Chinese elderly population (Shi et al., 2014).

### Anesthesia and surgery

Every patient undergoing total knee/hip arthroplasty under combined spinal and epidural anesthesia used the same surgical team to avoid the impact of different surgical techniques. Electrocardiogram, pulse oxygen saturation and non-invasive blood pressure were continuously monitored during anesthesia every 3 min. The spinal and epidural anesthesia was performed in the lateral decubitus under L_3–4_ space after the preparation was completed. And then 0.67% ropivacaine 2.0–2.5 ml was injected into the subarachnoid space after a successful puncture, afterward, 3–5 ml 2% lidocaine was given into the epidural catheter on account of actual needs to maintain the level of anesthesia at T_8_-S_5_. Intravenous ephedrine 6 mg was given if the intraoperative systolic blood pressure of the patient was < 90 mmHg;An intravenous injection of atropine 0.5 mg was given if the patient’s heart rate was < 50 bpm. All patients received patient-controlled intravenous analgesia (PCIA) for 48 h after operation. PCIA opioids consisted of 2.5 μg⋅kg^–1^ sufentanil and 5 mg tropisetron (total volume of 100 ml, including 0.9% normal saline, bolus 2 ml, basal rate 2 ml/h, locking time 15 min). Unless there was a clinical indication, postoperative analgesia was limited to non-opioid drugs. All clinical care details were recorded in case report forms. Midazolam injection and pumping into dexmetopramine were not allowed during the operation.

### Sample collection

Fasting lumbar CSF samples were collected and preserved in Qingdao Municipal Hospital Affiliated to Qingdao University. The CSF samples were taken when the spinal needle was first inserted through the Touhy needle and entered the thecal sac, before spinal medication given. The CSF samples were processed immediately within 2 h after standard lumbar puncture. They were centrifuged at 2,000 × g for 10 min, as well as separated and stored in an enzyme-free EP (Eppendorf) tube (oxygen bottle, PCR-02-C) at -80^°^C for further use in the following steps of this study. These samples were subjected to at most two freeze-thaw cycles.

### Enzyme-linked immunosorbent assay

CSF α-syn and core biomarkers (Aβ40, Aβ42, P-tau, T-tau), were measured by enzyme-linked immunosorbent assay (ELISA) and mini-tablet reader (Thermo Science Multiskan MK3). CSF α-syn was determined by ELISA kit (Legend Max human alpha synuclein ELISA kit pre-coated plate, catalog number: 844,101), and CSF core biomarkers (Aβ40, Aβ42, P-tau, T-tau) were determined by other ELISA kit (INNOTEST; Fujirebio). All ELISA tests were carried out by experienced technicians in strict accordance with the manufacturer’s instructions. They were blinded to the clinical information. The samples and standards were measured in duplicate, and the means of duplicates were used for statistical analyses. All antibodies and plates came from the same batch to rule out variability between batches. Intra-batch CV was < 5%, and inter-batch CV was < 15%.

### Sample size

The preliminary test in this study found that 8 co-variants (age, gender, education level, α-syn, Aβ40, Aβ42, P-tau, T-tau) were expected to enter the Logistic regression, and the preliminary test POD incidence was 12%, and the loss of follow-up rate was assumed to be 20%, so the required sample size was calculated to be 833 cases (8 × 10÷0.12÷0.8 = 833).

### Statistical analysis

Characteristics of the participants were represented as the mean ± standard deviation (SD), the median and quartile range (IQR, 25–75 percentiles), or a percentage (%). And the incidence of POD was expressed as a percentage. We used the Kolmogorov-Smirnov test to test the normality of all variables. Chi-square test was used to compare categorical variables. When the continuous variables were non-normal distribution, non-parametric methods were adopted. Mann-Whitney *U*-test was used to compare the difference between the two groups.

Binary logistic regression was used to analyze the associations between CSF α-syn and other biomarkers with POD. Co-variants included age, gender and education level as adjusted factors in multiple logistic regression. Multiple linear regression was used to analyze the associations between CSF α-syn and other biomarkers. Sensitivity analyses were performed by (1) adding more co-variants, including body weight, MMSE, MoCA, cigarette use (yes or no), alcohol intake (yes or no), diabetes mellitus (yes or no), hypertension (yes or no), coronary heart disease (yes or no), which showing the results were barely changed in this analysis; and (2) Screening patients with MMSE ≥ 28 and age from 65 to 90 years. Subgroup analyses were performed stratified by gender. *P* < 0.05 was statistically significant.

In order to explore whether the relationship between CSF α-syn and POD was mediated by POD pathology, the mediated analysis was fitted according to the method proposed by Baron and Kenny. The significance was determined by 5,000 bootstrap iterations using the mediation effect. The indirect effect (IE) was *P* < 0.05, which was considered to be significant.

The data were analyzed using SPSS version 23.0 (SPSS, Inc., Chicago, Illinois, USA), GraphPad Prism version 8.0 (GraphPad Software, Inc., LaJolla, CA, USA) and Stata MP 16.0 (Solvusoft Corporation, Inc., Chicago, Illinois, USA).

## Results

### Participant characteristics

**FIGURE 1:**
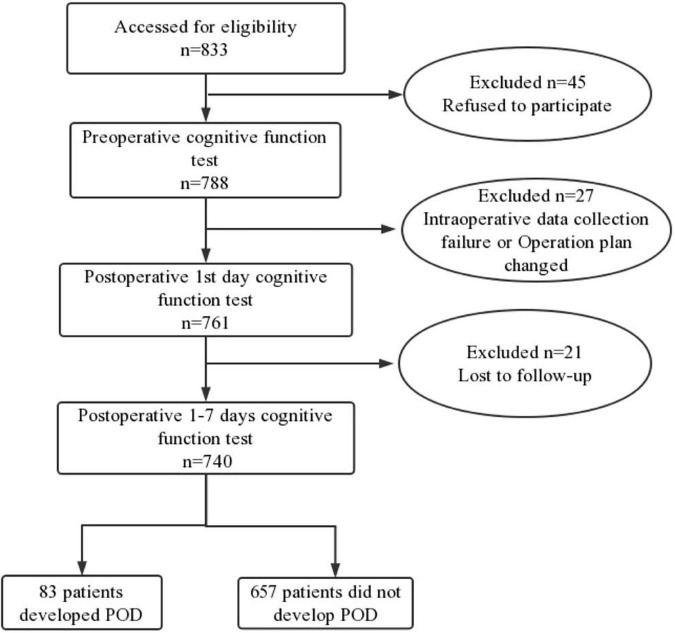
Flow diagram showed selection of eligible patients and the enrollment process. POD, postoperative delirium.

We included 833 participants, of which 740 met the requirements of this study and 155 participants were excluded. The reasons for dropping out were shown in Figure 1. Of the enrolled patients, 83 subjects experienced POD within 7 days after operation or before discharge, the demographic and clinical data of the participants were summarized in Table 1.

**TABLE 1:** Characteristics of participants.

	POD (*n* = 83)	Non-POD (*n* = 657)	*P*-values
Gender (Male/female)	46/37	395/262	0.411
Age (year), (mean ± *SD*)	62.5 ± 10.7	63.1 ± 10.7	0.604
Body weight (kg), **(Median, IQR)**	70 (65–75)	70 (65–78)	0.677
Education years, *n* (%)			0.168
0	6 (7.2)	33 (5.1)	0.149
1–9	48 (57.8)	351 (53.4)	
10–13	17 (20.5)	144 (21.9)	
14–17	12 (14.5)	129 (19.6)	
**ASA grade, *n* (%)**			
I	12 (14.5)	106 (16.1)	0.374
II	71 (85.5)	551 (83.9)	
MMSE, (Median, IQR)	28 (26–29)	28 (26–29)	
MoCA, (Median, IQR)	23 (20–26)	25 (21–28)	0.050
Cigarette use, yes (%)	26 (31.3)	202 (30.7)	0.914
Alcohol intake, yes, (%)	23 (27.7)	206 (31.4)	0.499
Hypertension, yes (%)	38 (45.8)	252 (38.3)	0.192
Diabetes mellitus, yes(%)	15 (18.1)	107 (16.3)	0.679
CHD, yes (%)	9 (10.8)	93 (14.2)	0.410
Duration of surgery (min) (Median, IQR)	140 (130–150)	140 (125–154)	0.777
Duration of anesthesia (min) (Median, IQR)	160 (150–170)	160 (45–174)	0.776
Intraoperative blood loss (ml) (Median, IQR)	30 (20–100)	50 (20–150)	0.064
NRS scores (Median, IQR)	2 (2–3)	3 (1–3)	0.050
MDAS scores	6 (5–8)	1 (0–2)	<0.001

Categorical variables are reported as numbers and percentages; continuous variables are reported as means ± SDs, whereas non-normal data are expressed as the median (IQR). The length of anesthesia was defined from the time when the anesthesiologists started the spinal anesthesia in the patients to the time when the patients were sent to the post-anesthesia care unit. The length of surgery was defined from the time of initial incision to the time of the closure of the skin.

POD, postoperative delirium; ASA, American Society of Anesthesiologists; min, minute; kg, kilogram; ml, milliliter; SD, standard deviation; CSF, cerebrospinal fluid; CHD, coronary heart disease; MMSE, Mini-Mental State Examination; MoCA, Montreal cognitive assessment; NRS, Numerical Rating Scale; MDAS, Memorial Delirium Assessment Scale.

The incidence of POD was 11.22% (83 of 740 patients). Of the 83 patients with POD, 46 patients were male. Compared with non-POD participants, there was no significant statistical difference in the baseline characteristic data of POD participants (*P* < 0.05). And MDAS score [1 (0ere was no significant statistical difference ifrom the patients who were diagnosed as POD [6 (5atist*P* < 0.001].

### The relationships of α-syn with cerebrospinal fluid biomarkers

**FIGURE 2:**
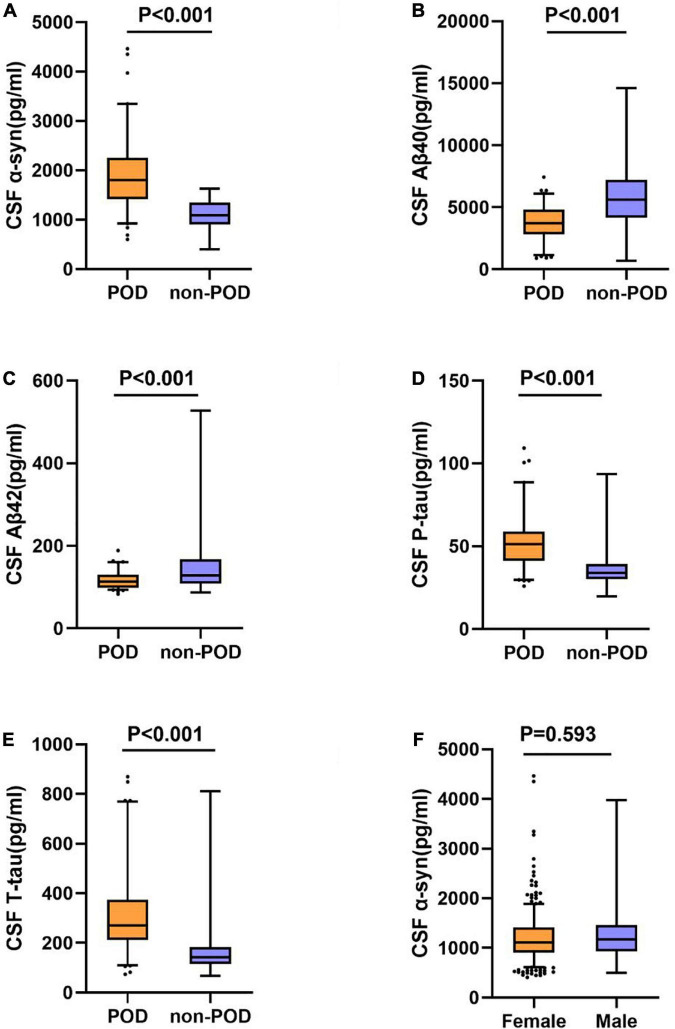
Expression ofα-syn and biomarkers in CSF of POD patients and non-POD controls. The scatter plots showed the expression levels of α-syn **(A)**, Aβ40 **(B)**, Aβ42 **(C)**, P-tau **(D)**, and T-tau **(E)**. CSF α-syn levels are independent of gender **(F)**. The colors of scatter maps are grouped according to different diagnostic groups. The *P*-value was determined by Mann-Whitney *U*-test. The contents of CSF biomarkers in patients with POD (POD group) were significantly different than those in patients without POD (non-POD group) (*P* < 0.001). POD, postoperative delirium; CSF, cerebrospinal fluid.

The concentrations of CSF α-syn and core biomarkers (Aβ40, Aβ42, T-tau, P-tau) were compared between POD patients and non-POD patients before operation. Mann-Whitney test showed that POD patients had higher levels of CSF α-syn than non-POD patients (Figure 2A). In addition, the CSF levels of P-tau and T-tau in patients with delirium were significantly higher than those in patients without delirium (Figures 2D,E). However, the CSF levels of Aβ40 and Aβ42 in POD patients were significantly lower than those in non-POD patients (Figures 2B,C).

The results indicated that CSF α-syn concentrations were not affected by gender [male = 1111.6 (910.8–1415.2) pg/ml, *n* = 441; female = 1173.5 (932.6–1460.8) pg/ml, *n* = 299; *P* = 0.593] (Figure 2F).

### The associations of cerebrospinal fluid biomarkers with postoperative delirium

The associations of CSF biomarkers with POD patients were shown in Table 2. Univariate binary logistic regression analysis showed that increased concentration of CSF α-syn (OR = 1.005, 95% CI 1.004–1.006, *P* < 0.001), P-tau (OR = 1.091, 95% CI 1.070–1.113, *P* < 0.001), T-tau (OR = 1.007, 95% CI 1.006–1.009, *P* < 0.001) were risk factors for POD. However, the increased concentration of Aβ42 (OR = 0.980, 95%CI 0.972–0.989, *P* < 0.001) was the protective factor of POD.

**TABLE 2:** Logistic regression analysis.

	Unadjusted				Adjusted	
	OR	95%CI	*P*-value	OR	95%CI	*P*-value
α-syn (pg/ml)	1.005	1.004–1.006	<0.001	1.005	1.004–1.006	<0.001
Aβ40 (pg/ml)	0.999	0.999–1.000	>0.05	–	–	–
Aβ42 (pg/ml)	0.980	0.972–0.989	<0.001	0.980	0.972–0.989	<0.001
P-tau (pg/ml)	1.091	1.070–1.113	<0.001	1.093	1.071–1.115	<0.001
T-tau (pg/ml)	1.007	1.006–1.009	<0.001	1.008	1.006–1.009	<0.001

The factors of postoperative delirium (POD) were assessed using binary logistic regression analysis adjusted age, gender and education level.

OR, odds ratio; CI, confidence interval.

In addition, after adjusting for age, gender, education level, increased CSF concentrations of α-syn (OR = 1.005, 95%CI 1.004–1.006, *P* < 0.001), P-tau (OR = 1.093, 95%CI 1.071–1.115, *P* < 0.001), T-tau (OR = 1.008, 95%CI 1.006–1.009, *P* < 0.001) were still the risk factors of POD. As expected, the increased concentration of Aβ42 (OR = 0.980, 95%CI 0.972–0.989, *P* < 0.001) was still the protective factor of POD.

### The relationships of α-syn and cerebrospinal fluid biomarkers

**FIGURE 3:**
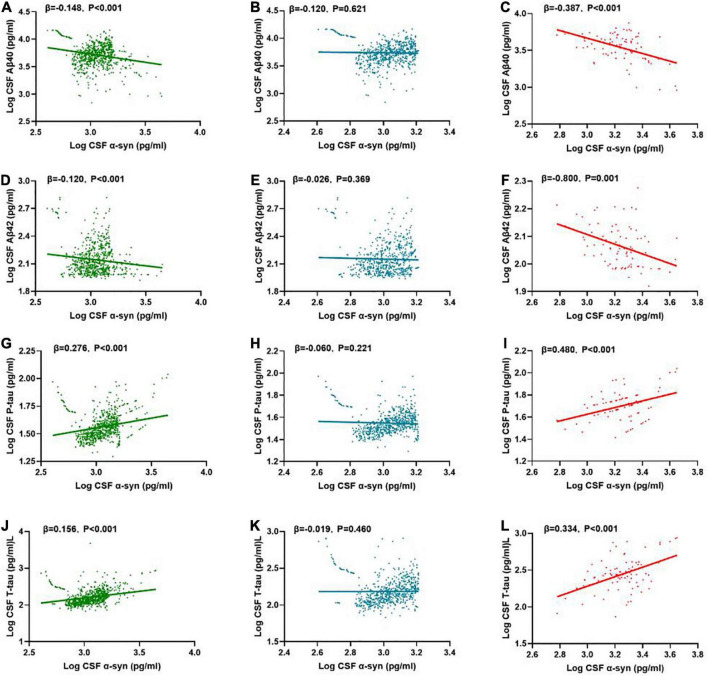
Associations of CSF α-syn and CSF core biomarkers. Scatter plots represent the associations of CSF α-syn with CSF core biomarkers: Aβ40, Aβ42, T-tau, P-tau in the different groups [whole cohort **(A,D,G,J)**, POD **(C,F,I,L)**, non-POD **(B,E,H,K)**]. The normalized regression coefficients (β) and *P*-values computed by multiple linear regression after adjustment for age, gender, educational level were shown. CSF, cerebrospinal fluid; α-syn, α-synuclein; Aβ40, amyloid-β40; Aβ42, amyloid-β42; T-tau, total Tau; P-tau, phosphorylated Tau.

In the multiple linear regression model adjusted for age, gender and education level, the correlations between α-syn and CSF biomarkers were tested. In the total participants (*n* = 740), CSF α-syn level was negatively correlated with Aβ40 (β = -0.148, *P* < 0.001), Aβ42 (β = -0.120, *P* < 0.001), while positively with P-tau (β = 0.276, *P* < 0.001) and T-tau (β = 0.156, *P* < 0.001) (Figures 3A,D,G,J).

In the non-POD group, CSF α-syn level was positively correlated with Aβ40 (β = -0.120, *P* = 0.621) (Figure 3C), Aβ42 (β = -0.026, *P* = 0.369), P-tau (β = -0.060, *P* = 0.221), and T-tau (β = -0.091, *P* = 0.460) (Figures 3B,E,H,K).

In the POD group, CSF α-syn level was negatively correlated with Aβ40 (β = -0.387, *P* < 0.001), Aβ42 (β = -0.800, *P* = 0.001), while positively with P-tau (β = 0.480, *P* < 0.001) (Figure 3H) and T-tau (β = 0.334, *P* < 0.001) (Figures 3C,F,I,L).

### Mediation analyses

**FIGURE 4:**
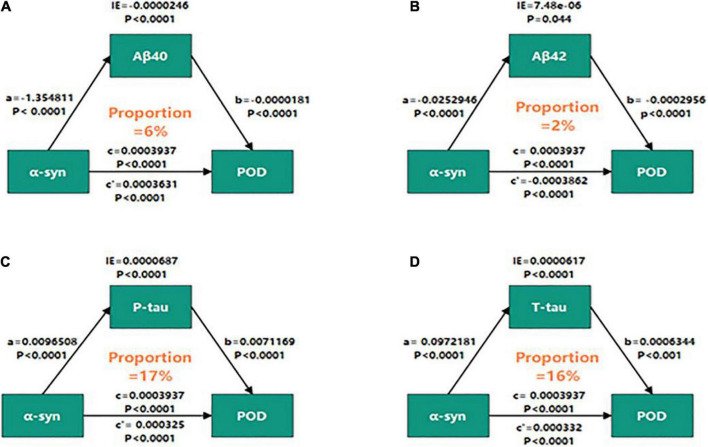
Mediation analyses with 5,000 bootstrapped iterations were used to examine mediation effects of Aβ40 **(A)**, Aβ42 **(B)**, P-tau **(C)**, and T-tau **(D)** on POD. CSF, cerebrospinal fluid; IE, indirect effect; α-syn, α-synuclein; Aβ40, amyloid-β40; Aβ42, amyloid-β42; T-tau, total Tau; P-tau, phosphorylated Tau.

Mediation analyses showed that the relationship between α-syn and POD was partly mediated by tau pathologies (the proportion of intermediaries is about 16–17%) (Figures 4C,D), independently of amyloid pathology (Figures 4A,B).

### Sensitivity analysis

The results were barely changed except that Aβ42 was not the protective factor of POD by (1) adding more covariates, including body weight, MMSE, MoCA, cigarette use (yes or no), alcohol intake (yes or no), diabetes mellitus (yes or no), hypertension (yes or no), coronary heart disease (yes or no), which showing the results were barely changed in this analysis; and (2) Screening patients with MMSE ≥ 28 and age from 65 to 90 years (OR = 0.983, 95% CI 0.967∼1.000, *P* = 0.05).

## Discussion

In this large prospective observational cohort study, we found that CSF α-syn is one of the preoperative risk factors for POD, which may be mediated through tau pathologies. The incidence of POD was 11.22%, which was almost consistent with the previous studies (Yang et al., 2017).

α-syn was a protein consisting of 140 amino acids, which mostly localizes to presynaptic terminals (Dułak et al., 2020). The injection of recombinant α-syn into wild-type mice could cause α-syn-containing inclusion bodies and brain dysfunction (Luk et al., 2012). A recent study suggested that increased plasma α-syn levels in patients with Parkinson’s disease (PD) were associated with cognitive impairment (Aarsland et al., 2017). And significant associations between POD and PD-related non-motor symptoms, which might impose a burden on α-syn deposition (Kim et al., 2018). The above evidences strongly suggested that POD represented the preclinical stage of α-syn-related cognitive impairment. Consistent with this, we observed that CSF α-syn level was an independent risk factor for POD and higher in POD patients before operation.

However, the relationship of CSF α-syn and POD and their mechanisms are still not clear. Meanwhile, although a kind of study in the biomarkers of neuroinflammation and synaptic dysfunction with POD is limited, accumulating evidence has shown that pathological α-syn, Aβ and Tau have synergistic adverse effects, promoting the aggregation of each other and subsequently amplifying neuronal damage (Giasson et al., 2003; Lee et al., 2004; Clinton et al., 2010; Nemani et al., 2010; Resende et al., 2013; Toledo et al., 2013; Slaets et al., 2014; Dolatshahi et al., 2018). α-syn plays a role in aggravating cognitive impairment. The increased CSF α-syn level in patients with cognitive dysfunction has been shown to reflect the increase in the α-syn level in brain tissue, which means that the CSF α-syn level may be related to the severity or progression of cognitive dysfunction (Giasson et al., 2003; Scott et al., 2010; Larson et al., 2012). α-syn could affect biological pathways and promote the formation of Aβ aggregates (Sidhu et al., 2004). In the case of neuronal injury, both aggregated Aβ and α-syn may be attached to synaptic membranes. Binding of α-syn to synaptic membranes can induce cytoplasmic α-syn to aggregate in the form of intracellular Lewy bodies, and to interact with the membrane-related peptides Aβ40 and Aβ42 (Mandal et al., 2006).

Our results showed the CSF levels of Aβ40 and Aβ42 in POD patients were significantly lower than those in non-POD patients, whereas the CSF levels of P-tau and T-tau in patients with delirium were significantly higher than those in patients without delirium, which suggested that P-tau and T-tau were risk factors of POD and Aβ40 and Aβ42 were protective factors. However, further analysis showed the Aβ40 and Aβ42 were not protective factors for POD, which is not consistent with the previous study (Cunningham et al., 2019) and maybe require more studies and further validation of a large sample. Meanwhile, our findings of this study showed that higher CSF α-syn concentrations were associated with tau pathologies (defined as elevated CSF P-tau or T-tau), while in the absence of Aβ pathology, as indicated by the differences between non-POD and POD individuals, Which suggested that the levels of increased CSF α-syn and tau pathologies, rather than Aβ pathology, related to POD may be interactional.

Over the past few years, there has been increasing evidence that tau and α-syn overlap in neuropathology, and their interactions compound to promote the progression of the disease (Galpern and Lang, 2006; Clinton et al., 2010). The interaction between α-syn and tau can lead to destruction of cytoskeleton tissue, defect of axonal transport and abnormality of synaptic tissue, which leads to cognitive-related neuronal dysfunction and death (Arai et al., 2001; Roy and Jackson, 2014). It is reported that in the presence of tau, α-syn aggregates at low concentrations and forms high molecular weight species (Badiola et al., 2011). In addition, the overexpression of tau also increased the secretion of α-syn and was related to delirium incidence and severity (Clarimón et al., 2009; Ballweg et al., 2021). Therefore, above researches supported our findings that the relationship between α-syn and POD was partly mediated by tau pathologies (the proportions of intermediaries were about 16–17%), independently of amyloid pathology.

The advantage of this current study is that a large number of subjects with good characteristics and biomarkers evidence of POD. In addition, we first explored the association between the expression level of α-syn in CSF and POD and used mediating effect to analyze the biomarkers in CSF mediate the relationship between α-syn and POD.

Our study also had some limitations. First of all, we did not screen the subjects for possible α-syn mutations. Although the prevalence of α-syn mutations in the general population or patients with cognitive dysfunction was very low, further study still would be needed. Secondly, we only explored the relationship between CSF α-syn and Aβ40, Aβ42, T-tau, P-tau in POD, other possible mechanism between CSF α-syn and POD needs to be further studied. Thirdly, the study without information regarding participant’s muscle mass, body mass index (sarcopenia and obesity) and functional status before operation, which will be taken in the next step in our research. Fourth, we calculate the sample size by the preliminary test in this study, however, the incidence of POD was 11.22% lower than the preliminary test (12%) at last, so the sample size is underpowered.

## Conclusion

In conclusion, CSF α-syn is one of the preoperative risk factors for POD, which may be mediated through tau pathologies. Our results confirm that α-syn would become a powerful predictor for POD. Furthermore, the possible mechanism-based studies are warranted.

## Data availability statement

The original contributions presented in this study are included in the article/supplementary material, further inquiries can be directed to the corresponding author/s.

## Ethics statement

The studies involving human participants were reviewed and approved by the Ethical Committee Qingdao Municipal Hospital affiliated to Qingdao University. The patients/participants provided their written informed consent to participate in this study. Written informed consent was obtained from the individual(s) for the publication of any potentially identifiable images or data included in this article.

## Author contributions

XL contributed to the study design, data collection, statistical analysis, and manuscript preparation. YG performed the ELISA. BW were involved in the data collection. RD performed the neuropsychological testing. YB contributed to the study concept and design, as well as manuscript preparation and review. XL and YG performed the statistical analysis. All authors contributed to the article and approved the submitted version.
